# Polymorph of 4-(carbazol-9-yl)benzo­nitrile

**DOI:** 10.1107/S1600536812012457

**Published:** 2012-03-28

**Authors:** Yu-Zhong Xie, Jing-Yi Jin, Xiao-Chun Qu

**Affiliations:** a Department of Chemistry, Yanbian University, Yanji Jilin 133002, People’s Republic of China

## Abstract

The asymmetric unit of the title compound, C_19_H_12_N_2_, contains two independent mol­ecules with a similar structure. In the two mol­ecules, the dihedral angles between the carbazole ring system and the benzene ring are 47.9 (5) and 45.4 (4)°, similar to the value of 47.89 (6)° found in the previously reported structure [Saha & Samanta (1999 ▶). *Acta Cryst.* C**55**, 1299–1300]. In the crystal, there is a weak C—H⋯N hydrogen bond between the two independent mol­ecules.

## Related literature

For related literature on intra­molecular charge transfer in electron donor–acceptor mol­ecules, see: Samanta *et al.* (2001 ▶); Galievsky *et al.* (2010 ▶); Megerle *et al.*(2008 ▶). For the previously reported structure of the title compound, see: Saha & Samanta (1999 ▶).
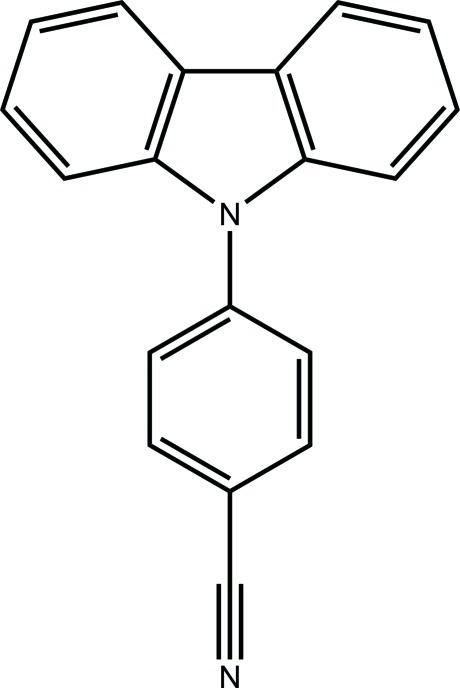


## Experimental

### 

#### Crystal data

C_19_H_12_N_2_
*M* *_r_* = 268.31
Monoclinic, 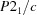

*a* = 15.5780 (17) Å
*b* = 8.054 (3) Å
*c* = 23.078 (5) Å
β = 93.088 (3)°
*V* = 2891.3 (14) Å^3^
*Z* = 8
Mo *K*α radiation
μ = 0.07 mm^−1^
*T* = 293 K
0.42 × 0.24 × 0.20 mm

#### Data collection

Bruker SMART APEXII CCD area-detector diffractometer
13972 measured reflections
5092 independent reflections
2310 reflections with *I* > 2σ(*I*)
*R*_int_ = 0.059

#### Refinement

*R*[*F* ^2^ > 2σ(*F* ^2^)] = 0.057
*wR*(*F* ^2^) = 0.192
*S* = 0.97
5092 reflections
380 parameters
H-atom parameters constrained
Δρ_max_ = 0.43 e Å^−3^
Δρ_min_ = −0.18 e Å^−3^

### 

Data collection: *APEX2* (Bruker, 2007 ▶); cell refinement: *SAINT* (Bruker, 2007 ▶); data reduction: *SAINT*; program(s) used to solve structure: *SHELXTL* (Sheldrick, 2008 ▶); program(s) used to refine structure: *SHELXTL*; molecular graphics: *SHELXTL*; software used to prepare material for publication: *SHELXTL*.

## Supplementary Material

Crystal structure: contains datablock(s) global, I. DOI: 10.1107/S1600536812012457/xu5478sup1.cif

Supplementary material file. DOI: 10.1107/S1600536812012457/xu5478Isup2.cdx

Structure factors: contains datablock(s) I. DOI: 10.1107/S1600536812012457/xu5478Isup3.hkl

Supplementary material file. DOI: 10.1107/S1600536812012457/xu5478Isup4.cml

Additional supplementary materials:  crystallographic information; 3D view; checkCIF report

## Figures and Tables

**Table 1:** Hydrogen-bond geometry (Å, °)

*D*—H⋯*A*	*D*—H	H⋯*A*	*D*⋯*A*	*D*—H⋯*A*
C17—H17⋯N3^i^	0.93	2.50	3.387 (6)	159

Symmetry code: (i) 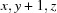.

**Fig. 1.**
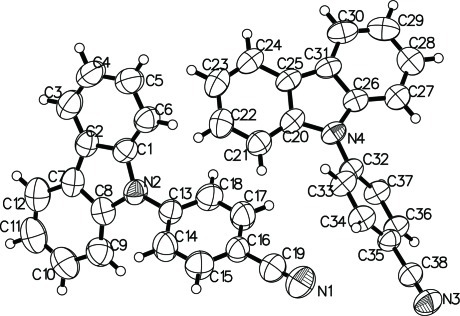
The molecular structure with the atom-numbering scheme. Displacement ellipsoids are drawn at 30% probability level..

## supplementary crystallographic information

### Comment

9-(4-cyanophenyl)carbazole, as an important compound with intramolecular charge transfer in electron donor-acceptor molecules has been a topic of extensive investigation in recent years (Samanta *et al.*, 2001; Galievsky *et al.*, 2010). The title compound, displays a single fluorescence band that has been tentatively assigned as an emission from the TICT state (Megerle *et al.*, 2008).

Since the ground-state structure of a system often determines the excited-state conformation of the molecule. The asymmetric unit of the title compound contains two molecule; the torsion angle is similar to that in the previously reported structure (Saha & Samanta, 1999).

### Experimental

The title compound was synthesized, according to the literature method (Saha & Samanta, 1999). A mixture of carbazole (5 g) and sodium hydride (0.36 g) was stirred in dry dimethylformamide (50 ml) under a nitrogen atmosphere for 2 h. The sodium salt of carbazole formed was then heated at 393 K with 4-fluorobenzonitrile (1.8 g) and sodium iodide (2.3 g) for about 20 h. The product, along with unreacted reactants, was precipitated by adding water to the reaction mixture. Yellow crystals were obtained from absolute chloroform upon slow evaporation of the solvent.

### Refinement

H atoms were positioned geometrically and refined using a riding model with C—H = 0.93 and with U_iso_(H) = 1.2U_eq_(C).

### Crystal data

**Table:**

C_19_H_12_N_2_	*F*(000) = 1120
*M**_r_* = 268.31	*D*_x_ = 1.233 Mg m^−^^3^
Monoclinic, *P*2_1_/*c*	Mo *K*α radiation, λ = 0.71069 Å
Hall symbol: -P 2ybc	Cell parameters from 13972 reflections
*a* = 15.5780 (17) Å	θ = 1.3–25.3°
*b* = 8.054 (3) Å	µ = 0.07 mm^−^^1^
*c* = 23.078 (5) Å	*T* = 293 K
β = 93.088 (3)°	Yellow, block
*V* = 2891.3 (14) Å^3^	0.42 × 0.24 × 0.20 mm
*Z* = 8	

### Data collection

**Table:**

Bruker SMART APEXII CCD area-detector diffractometer	2310 reflections with *I* > 2σ(*I*)
Radiation source: fine-focus sealed tube	*R*_int_ = 0.059
Graphite monochromator	θ_max_ = 25.0°, θ_min_ = 1.3°
φ and ω scans	*h* = −14→18
13972 measured reflections	*k* = −9→9
5092 independent reflections	*l* = −27→27

### Refinement

**Table:**

Refinement on *F*^2^	Secondary atom site location: difference Fourier map
Least-squares matrix: full	Hydrogen site location: inferred from neighbouring sites
*R*[*F*^2^ > 2σ(*F*^2^)] = 0.057	H-atom parameters constrained
*wR*(*F*^2^) = 0.192	*w* = 1/[σ^2^(*F*_o_^2^) + (0.0955*P*)^2^] where *P* = (*F*_o_^2^ + 2*F*_c_^2^)/3
*S* = 0.97	(Δ/σ)_max_ = 0.001
5092 reflections	Δρ_max_ = 0.43 e Å^−^^3^
380 parameters	Δρ_min_ = −0.18 e Å^−^^3^
0 restraints	Extinction correction: *SHELXTL* (Sheldrick, 2008), Fc^*^=kFc[1+0.001xFc^2^λ^3^/sin(2θ)]^-1/4^
Primary atom site location: structure-invariant direct methods	Extinction coefficient: 0.0065 (11)

### Special details

**Table:**

Geometry. All s.u.'s (except the s.u. in the dihedral angle between two l.s. planes) are estimated using the full covariance matrix. The cell s.u.'s are taken into account individually in the estimation of s.u.'s in distances, angles and torsion angles; correlations between s.u.'s in cell parameters are only used when they are defined by crystal symmetry. An approximate (isotropic) treatment of cell s.u.'s is used for estimating s.u.'s involving l.s. planes.
Refinement. Refinement of *F*^2^ against ALL reflections. The weighted *R*-factor *wR* and goodness of fit *S* are based on *F*^2^, conventional *R*-factors *R* are based on *F*, with *F* set to zero for negative *F*^2^. The threshold expression of *F*^2^ > 2σ(*F*^2^) is used only for calculating *R*-factors(gt) *etc*. and is not relevant to the choice of reflections for refinement. *R*-factors based on *F*^2^ are statistically about twice as large as those based on *F*, and *R*- factors based on ALL data will be even larger.

### Fractional atomic coordinates and isotropic or equivalent isotropic displacement parameters (Å^2^)

**Table:**

	*x*	*y*	*z*	*U*_iso_*/*U*_eq_	
C1	0.0794 (2)	0.5654 (4)	0.17499 (15)	0.0637 (9)	
C2	0.0258 (2)	0.6956 (4)	0.18921 (16)	0.0673 (9)	
C3	0.0519 (3)	0.8042 (5)	0.23307 (18)	0.0868 (12)	
H3	0.0169	0.8921	0.2430	0.104*	
C4	0.1299 (3)	0.7801 (5)	0.26158 (18)	0.0924 (13)	
H4	0.1476	0.8523	0.2913	0.111*	
C5	0.1838 (3)	0.6496 (5)	0.24714 (16)	0.0849 (12)	
H5	0.2370	0.6369	0.2669	0.102*	
C6	0.1586 (2)	0.5398 (4)	0.20384 (16)	0.0756 (10)	
H6	0.1936	0.4514	0.1943	0.091*	
C7	−0.0514 (2)	0.6817 (4)	0.15240 (16)	0.0697 (10)	
C8	−0.0415 (2)	0.5434 (5)	0.11718 (16)	0.0703 (10)	
C9	−0.1045 (3)	0.4963 (5)	0.07474 (17)	0.0862 (12)	
H9	−0.0969	0.4044	0.0511	0.103*	
C10	−0.1774 (3)	0.5906 (6)	0.0694 (2)	0.1009 (14)	
H10	−0.2202	0.5617	0.0416	0.121*	
C11	−0.1896 (3)	0.7281 (6)	0.1041 (2)	0.1031 (15)	
H11	−0.2402	0.7890	0.0996	0.124*	
C12	−0.1266 (3)	0.7750 (5)	0.1455 (2)	0.0919 (13)	
H12	−0.1345	0.8679	0.1686	0.110*	
C13	0.0687 (2)	0.3197 (4)	0.10821 (14)	0.0626 (9)	
C14	0.0166 (3)	0.1809 (5)	0.10756 (18)	0.0881 (12)	
H14	−0.0386	0.1875	0.1208	0.106*	
C15	0.0473 (3)	0.0313 (5)	0.08692 (18)	0.0912 (12)	
H15	0.0122	−0.0621	0.0858	0.109*	
C16	0.1285 (2)	0.0206 (4)	0.06828 (15)	0.0703 (10)	
C17	0.1788 (3)	0.1584 (5)	0.06795 (18)	0.0899 (13)	
H17	0.2338	0.1512	0.0545	0.108*	
C18	0.1491 (3)	0.3089 (5)	0.08743 (17)	0.0847 (12)	
H18	0.1837	0.4028	0.0864	0.102*	
C19	0.1611 (3)	−0.1367 (6)	0.04958 (19)	0.0955 (13)	
C20	0.3968 (2)	0.0673 (4)	0.18137 (13)	0.0583 (9)	
C21	0.3155 (2)	0.0319 (4)	0.19946 (15)	0.0684 (9)	
H21	0.2838	−0.0573	0.1843	0.082*	
C22	0.2836 (3)	0.1345 (5)	0.24091 (16)	0.0791 (11)	
H22	0.2290	0.1142	0.2537	0.095*	
C23	0.3305 (3)	0.2667 (5)	0.26402 (16)	0.0812 (11)	
H23	0.3078	0.3311	0.2930	0.097*	
C24	0.4100 (3)	0.3044 (4)	0.24484 (14)	0.0706 (10)	
H24	0.4407	0.3949	0.2600	0.085*	
C25	0.4441 (2)	0.2052 (4)	0.20239 (13)	0.0581 (9)	
C26	0.5215 (2)	0.0739 (4)	0.13402 (13)	0.0564 (8)	
C27	0.5904 (2)	0.0393 (4)	0.10028 (14)	0.0654 (9)	
H27	0.5909	−0.0550	0.0770	0.078*	
C28	0.6580 (2)	0.1497 (5)	0.10251 (16)	0.0776 (11)	
H28	0.7049	0.1297	0.0802	0.093*	
C29	0.6576 (3)	0.2910 (5)	0.13759 (17)	0.0818 (11)	
H29	0.7033	0.3652	0.1375	0.098*	
C30	0.5904 (3)	0.3214 (5)	0.17211 (15)	0.0744 (11)	
H30	0.5910	0.4149	0.1957	0.089*	
C31	0.5218 (2)	0.2119 (4)	0.17159 (13)	0.0574 (8)	
C32	0.4241 (2)	−0.1677 (4)	0.11298 (13)	0.0557 (8)	
C33	0.3985 (2)	−0.3007 (4)	0.14558 (14)	0.0652 (9)	
H33	0.3923	−0.2882	0.1852	0.078*	
C34	0.3822 (2)	−0.4510 (4)	0.11962 (15)	0.0669 (9)	
H34	0.3643	−0.5400	0.1416	0.080*	
C35	0.39242 (19)	−0.4711 (4)	0.06052 (14)	0.0566 (8)	
C36	0.4157 (2)	−0.3374 (4)	0.02797 (14)	0.0624 (9)	
H36	0.4215	−0.3497	−0.0117	0.075*	
C37	0.4304 (2)	−0.1855 (4)	0.05374 (13)	0.0608 (9)	
H37	0.4446	−0.0946	0.0313	0.073*	
C38	0.3810 (2)	−0.6316 (5)	0.03491 (15)	0.0669 (10)	
N1	0.1879 (3)	−0.2599 (5)	0.0349 (2)	0.1307 (16)	
N2	0.03835 (18)	0.4708 (3)	0.13070 (12)	0.0674 (8)	
N3	0.3724 (2)	−0.7593 (4)	0.01431 (15)	0.0902 (10)	
N4	0.44459 (17)	−0.0136 (3)	0.13990 (11)	0.0588 (7)	

### Atomic displacement parameters (Å^2^)

**Table:**

	*U*^11^	*U*^22^	*U*^33^	*U*^12^	*U*^13^	*U*^23^
C1	0.077 (3)	0.051 (2)	0.064 (2)	−0.005 (2)	0.0096 (19)	0.0006 (18)
C2	0.079 (3)	0.051 (2)	0.073 (2)	0.000 (2)	0.020 (2)	0.001 (2)
C3	0.105 (3)	0.069 (3)	0.089 (3)	−0.002 (3)	0.025 (2)	−0.011 (2)
C4	0.117 (4)	0.078 (3)	0.084 (3)	−0.023 (3)	0.021 (3)	−0.022 (2)
C5	0.092 (3)	0.081 (3)	0.082 (3)	−0.012 (2)	0.004 (2)	−0.008 (2)
C6	0.084 (3)	0.061 (2)	0.083 (3)	−0.001 (2)	0.008 (2)	−0.007 (2)
C7	0.080 (3)	0.057 (2)	0.075 (2)	0.008 (2)	0.023 (2)	0.009 (2)
C8	0.069 (2)	0.067 (3)	0.075 (2)	0.005 (2)	0.012 (2)	0.012 (2)
C9	0.080 (3)	0.092 (3)	0.087 (3)	0.010 (2)	−0.001 (2)	0.004 (2)
C10	0.075 (3)	0.118 (4)	0.110 (4)	0.012 (3)	0.001 (2)	0.015 (3)
C11	0.086 (3)	0.102 (4)	0.123 (4)	0.024 (3)	0.022 (3)	0.023 (3)
C12	0.098 (3)	0.077 (3)	0.103 (3)	0.012 (3)	0.033 (3)	0.014 (2)
C13	0.068 (2)	0.054 (2)	0.067 (2)	−0.0026 (19)	0.0122 (18)	0.0004 (18)
C14	0.083 (3)	0.074 (3)	0.110 (3)	−0.005 (2)	0.032 (2)	−0.001 (2)
C15	0.100 (3)	0.061 (3)	0.114 (3)	−0.011 (2)	0.021 (3)	−0.001 (2)
C16	0.070 (3)	0.060 (3)	0.082 (2)	−0.002 (2)	0.0149 (19)	−0.006 (2)
C17	0.082 (3)	0.076 (3)	0.115 (3)	−0.008 (2)	0.030 (2)	−0.013 (3)
C18	0.095 (3)	0.061 (3)	0.100 (3)	−0.015 (2)	0.023 (2)	−0.009 (2)
C19	0.090 (3)	0.082 (3)	0.115 (3)	0.005 (3)	0.011 (3)	−0.021 (3)
C20	0.083 (3)	0.045 (2)	0.0468 (18)	0.0096 (18)	−0.0018 (17)	0.0031 (16)
C21	0.077 (3)	0.060 (2)	0.068 (2)	0.004 (2)	0.0014 (19)	−0.0002 (19)
C22	0.084 (3)	0.078 (3)	0.076 (3)	0.016 (2)	0.010 (2)	0.000 (2)
C23	0.102 (3)	0.073 (3)	0.068 (2)	0.024 (3)	0.006 (2)	−0.007 (2)
C24	0.098 (3)	0.056 (2)	0.057 (2)	0.010 (2)	−0.006 (2)	−0.0056 (18)
C25	0.080 (2)	0.048 (2)	0.0455 (18)	0.0069 (18)	−0.0099 (17)	0.0005 (16)
C26	0.070 (2)	0.051 (2)	0.0469 (18)	0.0002 (18)	−0.0045 (16)	0.0065 (16)
C27	0.077 (3)	0.064 (2)	0.055 (2)	0.002 (2)	0.0015 (18)	0.0061 (17)
C28	0.074 (3)	0.086 (3)	0.072 (2)	0.000 (2)	0.0035 (19)	0.017 (2)
C29	0.089 (3)	0.078 (3)	0.076 (3)	−0.023 (2)	−0.014 (2)	0.011 (2)
C30	0.095 (3)	0.066 (3)	0.061 (2)	−0.009 (2)	−0.016 (2)	0.0013 (19)
C31	0.075 (2)	0.047 (2)	0.0487 (18)	0.0009 (18)	−0.0127 (16)	0.0020 (16)
C32	0.071 (2)	0.043 (2)	0.0527 (19)	0.0051 (16)	−0.0028 (16)	0.0017 (16)
C33	0.090 (3)	0.053 (2)	0.0526 (19)	0.0001 (19)	0.0041 (17)	0.0024 (18)
C34	0.085 (3)	0.048 (2)	0.067 (2)	−0.0002 (18)	0.0015 (18)	0.0111 (18)
C35	0.062 (2)	0.045 (2)	0.062 (2)	0.0046 (16)	−0.0030 (16)	−0.0049 (17)
C36	0.076 (2)	0.059 (2)	0.0518 (19)	−0.0027 (18)	0.0004 (16)	−0.0032 (18)
C37	0.080 (2)	0.050 (2)	0.053 (2)	−0.0059 (17)	0.0024 (16)	0.0046 (17)
C38	0.070 (2)	0.055 (2)	0.077 (2)	−0.0006 (19)	0.0055 (18)	−0.008 (2)
N1	0.121 (3)	0.091 (3)	0.180 (4)	0.017 (3)	0.007 (3)	−0.047 (3)
N2	0.071 (2)	0.0565 (19)	0.0748 (19)	0.0104 (16)	0.0008 (15)	−0.0044 (16)
N3	0.102 (3)	0.063 (2)	0.107 (3)	−0.0088 (19)	0.012 (2)	−0.022 (2)
N4	0.0781 (19)	0.0447 (17)	0.0534 (16)	−0.0023 (14)	0.0017 (14)	−0.0030 (13)

### Geometric parameters (Å, º)

**Table:**

C1—C6	1.386 (5)	C20—C21	1.384 (4)
C1—C2	1.391 (4)	C20—N4	1.404 (4)
C1—N2	1.401 (4)	C20—C25	1.405 (4)
C2—C3	1.382 (5)	C21—C22	1.377 (4)
C2—C7	1.438 (5)	C21—H21	0.9300
C3—C4	1.365 (6)	C22—C23	1.382 (5)
C3—H3	0.9300	C22—H22	0.9300
C4—C5	1.396 (5)	C23—C24	1.371 (5)
C4—H4	0.9300	C23—H23	0.9300
C5—C6	1.376 (5)	C24—C25	1.391 (4)
C5—H5	0.9300	C24—H24	0.9300
C6—H6	0.9300	C25—C31	1.437 (5)
C7—C8	1.392 (5)	C26—C27	1.389 (4)
C7—C12	1.394 (5)	C26—N4	1.402 (4)
C8—N2	1.395 (4)	C26—C31	1.409 (4)
C8—C9	1.401 (5)	C27—C28	1.376 (5)
C9—C10	1.367 (5)	C27—H27	0.9300
C9—H9	0.9300	C28—C29	1.397 (5)
C10—C11	1.386 (6)	C28—H28	0.9300
C10—H10	0.9300	C29—C30	1.370 (5)
C11—C12	1.384 (6)	C29—H29	0.9300
C11—H11	0.9300	C30—C31	1.386 (5)
C12—H12	0.9300	C30—H30	0.9300
C13—C18	1.368 (5)	C32—C33	1.381 (4)
C13—C14	1.380 (5)	C32—C37	1.383 (4)
C13—N2	1.414 (4)	C32—N4	1.416 (4)
C14—C15	1.390 (5)	C33—C34	1.368 (4)
C14—H14	0.9300	C33—H33	0.9300
C15—C16	1.360 (5)	C34—C35	1.391 (4)
C15—H15	0.9300	C34—H34	0.9300
C16—C17	1.358 (5)	C35—C36	1.373 (4)
C16—C19	1.439 (6)	C35—C38	1.429 (5)
C17—C18	1.381 (5)	C36—C37	1.374 (4)
C17—H17	0.9300	C36—H36	0.9300
C18—H18	0.9300	C37—H37	0.9300
C19—N1	1.136 (5)	C38—N3	1.138 (4)
			
C6—C1—C2	122.0 (3)	C22—C21—C20	117.1 (3)
C6—C1—N2	129.0 (3)	C22—C21—H21	121.4
C2—C1—N2	109.0 (3)	C20—C21—H21	121.4
C3—C2—C1	119.5 (4)	C21—C22—C23	121.9 (4)
C3—C2—C7	133.3 (4)	C21—C22—H22	119.1
C1—C2—C7	107.2 (3)	C23—C22—H22	119.1
C4—C3—C2	118.9 (4)	C24—C23—C22	120.9 (4)
C4—C3—H3	120.6	C24—C23—H23	119.6
C2—C3—H3	120.6	C22—C23—H23	119.6
C3—C4—C5	121.5 (4)	C23—C24—C25	119.1 (3)
C3—C4—H4	119.2	C23—C24—H24	120.5
C5—C4—H4	119.2	C25—C24—H24	120.5
C6—C5—C4	120.4 (4)	C24—C25—C20	119.0 (3)
C6—C5—H5	119.8	C24—C25—C31	133.6 (3)
C4—C5—H5	119.8	C20—C25—C31	107.4 (3)
C5—C6—C1	117.7 (4)	C27—C26—N4	130.1 (3)
C5—C6—H6	121.1	C27—C26—C31	121.7 (3)
C1—C6—H6	121.1	N4—C26—C31	108.2 (3)
C8—C7—C12	118.9 (4)	C28—C27—C26	117.5 (3)
C8—C7—C2	107.0 (3)	C28—C27—H27	121.2
C12—C7—C2	134.1 (4)	C26—C27—H27	121.2
C7—C8—N2	109.3 (3)	C27—C28—C29	121.4 (4)
C7—C8—C9	122.0 (4)	C27—C28—H28	119.3
N2—C8—C9	128.7 (4)	C29—C28—H28	119.3
C10—C9—C8	117.4 (4)	C30—C29—C28	120.6 (4)
C10—C9—H9	121.3	C30—C29—H29	119.7
C8—C9—H9	121.3	C28—C29—H29	119.7
C9—C10—C11	122.0 (4)	C29—C30—C31	119.6 (4)
C9—C10—H10	119.0	C29—C30—H30	120.2
C11—C10—H10	119.0	C31—C30—H30	120.2
C12—C11—C10	120.3 (4)	C30—C31—C26	119.0 (3)
C12—C11—H11	119.9	C30—C31—C25	133.6 (3)
C10—C11—H11	119.9	C26—C31—C25	107.4 (3)
C11—C12—C7	119.4 (4)	C33—C32—C37	119.7 (3)
C11—C12—H12	120.3	C33—C32—N4	120.4 (3)
C7—C12—H12	120.3	C37—C32—N4	119.9 (3)
C18—C13—C14	119.5 (3)	C34—C33—C32	120.0 (3)
C18—C13—N2	120.9 (3)	C34—C33—H33	120.0
C14—C13—N2	119.6 (3)	C32—C33—H33	120.0
C13—C14—C15	119.5 (4)	C33—C34—C35	120.3 (3)
C13—C14—H14	120.2	C33—C34—H34	119.9
C15—C14—H14	120.2	C35—C34—H34	119.9
C16—C15—C14	120.4 (4)	C36—C35—C34	119.5 (3)
C16—C15—H15	119.8	C36—C35—C38	120.9 (3)
C14—C15—H15	119.8	C34—C35—C38	119.5 (3)
C17—C16—C15	119.7 (4)	C35—C36—C37	120.3 (3)
C17—C16—C19	120.3 (4)	C35—C36—H36	119.9
C15—C16—C19	120.0 (4)	C37—C36—H36	119.9
C16—C17—C18	120.8 (4)	C36—C37—C32	120.1 (3)
C16—C17—H17	119.6	C36—C37—H37	120.0
C18—C17—H17	119.6	C32—C37—H37	120.0
C13—C18—C17	119.9 (4)	N3—C38—C35	179.5 (4)
C13—C18—H18	120.0	C8—N2—C1	107.5 (3)
C17—C18—H18	120.0	C8—N2—C13	126.2 (3)
N1—C19—C16	179.1 (5)	C1—N2—C13	126.0 (3)
C21—C20—N4	129.6 (3)	C26—N4—C20	108.6 (3)
C21—C20—C25	122.0 (3)	C26—N4—C32	124.9 (3)
N4—C20—C25	108.3 (3)	C20—N4—C32	126.2 (3)
			
C6—C1—C2—C3	0.5 (5)	C31—C26—C27—C28	−3.2 (5)
N2—C1—C2—C3	178.5 (3)	C26—C27—C28—C29	0.3 (5)
C6—C1—C2—C7	−178.1 (3)	C27—C28—C29—C30	1.7 (5)
N2—C1—C2—C7	−0.1 (4)	C28—C29—C30—C31	−0.9 (5)
C1—C2—C3—C4	−0.3 (5)	C29—C30—C31—C26	−1.9 (5)
C7—C2—C3—C4	177.9 (4)	C29—C30—C31—C25	178.6 (3)
C2—C3—C4—C5	0.5 (6)	C27—C26—C31—C30	4.1 (4)
C3—C4—C5—C6	−0.9 (6)	N4—C26—C31—C30	−177.9 (3)
C4—C5—C6—C1	1.0 (5)	C27—C26—C31—C25	−176.4 (3)
C2—C1—C6—C5	−0.8 (5)	N4—C26—C31—C25	1.7 (3)
N2—C1—C6—C5	−178.4 (3)	C24—C25—C31—C30	−1.5 (6)
C3—C2—C7—C8	−178.4 (4)	C20—C25—C31—C30	177.3 (3)
C1—C2—C7—C8	0.0 (4)	C24—C25—C31—C26	179.1 (3)
C3—C2—C7—C12	3.0 (7)	C20—C25—C31—C26	−2.2 (3)
C1—C2—C7—C12	−178.7 (4)	C37—C32—C33—C34	2.2 (5)
C12—C7—C8—N2	179.1 (3)	N4—C32—C33—C34	−177.3 (3)
C2—C7—C8—N2	0.2 (4)	C32—C33—C34—C35	0.8 (5)
C12—C7—C8—C9	0.6 (5)	C33—C34—C35—C36	−2.5 (5)
C2—C7—C8—C9	−178.3 (3)	C33—C34—C35—C38	175.7 (3)
C7—C8—C9—C10	−0.8 (5)	C34—C35—C36—C37	1.2 (5)
N2—C8—C9—C10	−178.9 (4)	C38—C35—C36—C37	−177.0 (3)
C8—C9—C10—C11	0.1 (6)	C35—C36—C37—C32	1.8 (5)
C9—C10—C11—C12	0.6 (7)	C33—C32—C37—C36	−3.5 (5)
C10—C11—C12—C7	−0.7 (6)	N4—C32—C37—C36	176.0 (3)
C8—C7—C12—C11	0.1 (6)	C36—C35—C38—N3	46 (62)
C2—C7—C12—C11	178.7 (4)	C34—C35—C38—N3	−132 (62)
C18—C13—C14—C15	1.3 (6)	C7—C8—N2—C1	−0.2 (4)
N2—C13—C14—C15	−178.2 (3)	C9—C8—N2—C1	178.1 (3)
C13—C14—C15—C16	1.0 (6)	C7—C8—N2—C13	173.8 (3)
C14—C15—C16—C17	−2.3 (6)	C9—C8—N2—C13	−7.9 (6)
C14—C15—C16—C19	177.0 (4)	C6—C1—N2—C8	178.0 (3)
C15—C16—C17—C18	1.3 (6)	C2—C1—N2—C8	0.2 (4)
C19—C16—C17—C18	−178.1 (4)	C6—C1—N2—C13	4.0 (5)
C14—C13—C18—C17	−2.3 (6)	C2—C1—N2—C13	−173.8 (3)
N2—C13—C18—C17	177.2 (3)	C18—C13—N2—C8	132.5 (4)
C16—C17—C18—C13	1.0 (6)	C14—C13—N2—C8	−48.0 (5)
C17—C16—C19—N1	17 (32)	C18—C13—N2—C1	−54.6 (5)
C15—C16—C19—N1	−163 (100)	C14—C13—N2—C1	124.9 (4)
N4—C20—C21—C22	−179.3 (3)	C27—C26—N4—C20	177.3 (3)
C25—C20—C21—C22	−2.4 (5)	C31—C26—N4—C20	−0.6 (3)
C20—C21—C22—C23	−0.4 (5)	C27—C26—N4—C32	3.6 (5)
C21—C22—C23—C24	2.3 (6)	C31—C26—N4—C32	−174.2 (3)
C22—C23—C24—C25	−1.4 (5)	C21—C20—N4—C26	176.4 (3)
C23—C24—C25—C20	−1.4 (5)	C25—C20—N4—C26	−0.8 (3)
C23—C24—C25—C31	177.3 (3)	C21—C20—N4—C32	−10.0 (5)
C21—C20—C25—C24	3.3 (4)	C25—C20—N4—C32	172.7 (3)
N4—C20—C25—C24	−179.2 (3)	C33—C32—N4—C26	127.1 (3)
C21—C20—C25—C31	−175.7 (3)	C37—C32—N4—C26	−52.4 (4)
N4—C20—C25—C31	1.9 (3)	C33—C32—N4—C20	−45.4 (4)
N4—C26—C27—C28	179.2 (3)	C37—C32—N4—C20	135.1 (3)

### Hydrogen-bond geometry (Å, º)

**Table:**

*D*—H···*A*	*D*—H	H···*A*	*D*···*A*	*D*—H···*A*
C17—H17···N3^i^	0.93	2.50	3.387 (6)	159

Symmetry code: (i) *x*, *y*+1, *z*.
